# Editorial: Stress in health and disease: new era and new challenges

**DOI:** 10.3389/fphys.2023.1303345

**Published:** 2023-10-13

**Authors:** Peter N. Uchakin, Sarah C. Hellewell, Raymond P. Stowe

**Affiliations:** ^1^ Department of Biomedical Sciences, Mercer University School of Medicine, Macon, GA, United States; ^2^ Department of Internal Medicine Sciences, Mercer University School of Medicine, Macon, GA, United States; ^3^ Curtin Health Innovation Research Institute, Curtin University, Bentley, WA, Australia; ^4^ Perron Institute for Neurological and Translational Science, Nedlands, WA, Australia; ^5^ Centre for Neuromuscular & Neurological Disorders, University of Western Australia, Crawley, WA, Australia; ^6^ Microgen Laboratories, La Marque, TX, United States

**Keywords:** stress, inflammation, cancer, PTSD, cannabidol, cardiovascular disease

**FIGURE 1:**
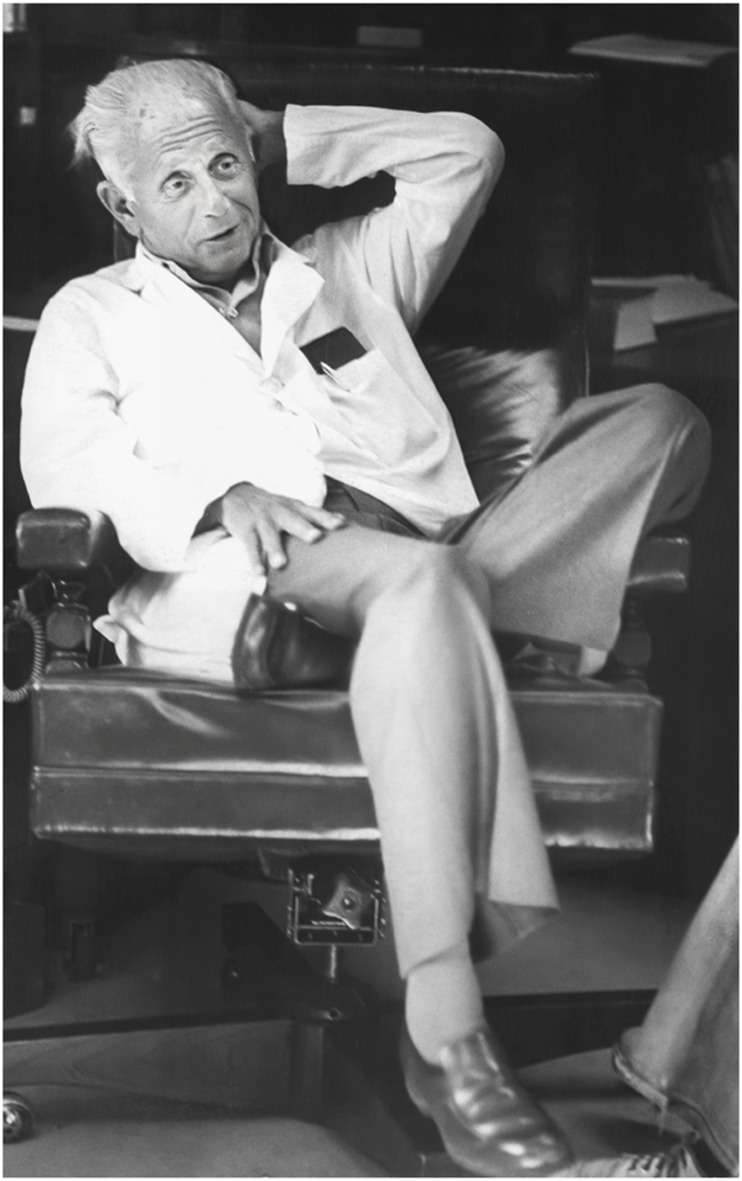
Photo of Dr. Hans Selye, pictured in 1977. Photo by Len Sidaway/Montreal Gazette files. Material republished with the express permission of Montreal Gazette, a division of Postmedia Network Inc. https://montrealgazette.com/opinion/columnists/the-right-chemistry-dr-hans-selye-and-the-syndrome-of-just-being-sick.

This Research Topic was dedicated to János (Hans) Selye, whom many consider to be the ‘father of stress research’. This Research Topic was launched in 2022, 40 years since the death of Selye and 86 years following the publication of his seminal manuscript “A Syndrome Produced by Diverse Nocuous Agents” in Nature (Selye, 1936). A prolific author, Selye’s publications numbered more than 1,700 scientific articles and approximately 40 books. He was a Nobel Prize nominee in 1949, the first of 17 such nominations in Physiology or Medicine. Though never ultimately awarded a Nobel, Selye’s work has been widely regarded as pioneering in the intellectual origins of biological stress, and a paradigm shift in the concept of a multi-system stress response. Selye founded the International Institute of Stress in 1975, the Hans Selye Foundation in 1979, and, with several other prominent stress researchers of the 1970s, the Canadian Institute of Stress (Figure 1).

Hans Selye dedicated his famous book ‘The Stress of Life’ to those “who are not afraid to enjoy the stress of a full life, nor too naïve to think that they can do so without intellectual effort” (Selye, 1956).

Perhaps, this reflects his emphasis on the need to consider all aspects of the full life with understanding that both good and bad are intertwined, and the importance of comprehending this fact in order to adequately counteract the negative aspects of the stress, and fully enjoy the positive ones. To wit, Selye’s statement in his classical book ‘Stress without Distress’, “Complete freedom from stress is death,” even further bonds stress and life, illustrating that life not only includes stressful situations, but that stress is woven through the fabric of life (Selye, 1974).

One may argue that stress is the basis of evolution, as it takes you out of your comfort zone, and to a place where learning occurs. And with learning comes adaptation, growth, and evolution. The simplest but perhaps the most common form of learning is sensory adaptation and habituation. In this Research Topic, Bullock et al. evaluate human adaptability to cold exposure with a wide range of objective as well subjective physiological markers: electrocardiography, impedance cardiography, blood pressure, pupillometry, scalp electroencephalography, salivary cortisol, and perception of the pain. In their extensive studies, they demonstrate dynamics of adaptation in these different markers, reflecting both sympathetic and parasympathetic responsiveness. Such studies are significant to better understanding the interactive nature of the physiological stress response, which Selye referred to as General Adaptation Syndrome.

The ability to cope with stressful factors is a life-preserving necessity. Use of chemicals, biologicals, or/and psychological countermeasures to support stress coping is a significant and growing part of healthcare as it helps to withstand negative effects of a stressful challenge. In particular, the use of cannabidiol-containing biological agents is increasing in popularity as the legalization of cannabis becomes more widespread and its healthcare benefits are increasingly investigated. In their studies, Hopkins et al. used cannabidiol extract to counteract stress-induced sterile inflammation in male and female rats. They showed sex- and tissue-dependent differences in immune stress reactivity and response to the oral treatment with cannabidiol extract, adding to a growing body of research demonstrating that cannabidiol has anti-inflammatory and anti-stress benefits.

Complex cognitive and non-cognitive chronic stressors of life may affect immune homeostasis via different pathways, of which the hypothalamic-pituitary-adrenal (HPA) axis is the most apparent and well characterized. Alterations in the HPA axis may lead to either 1) immunosenescence, with increased risk of infections and/or neoplasm; or 2) autoimmunity, which may lead the development of cardio-vascular, metabolic, or neurogenerative pathologies. Thus, in this Research Topic, we selected contributors that emphasize the link between stress and clinical outcomes.

The review by Petrinovic et al. provides the reader with an overview of how chronic stress and cancer have a bi-directional relationship, and demonstrates that low-grade inflammation is a common theme in this relationship. They further extensively discuss biological mechanisms of such a relationship, as well as possible ways of targeting inflammation in the prevention and treatment of cancer. In the next selected review, Slusher et al. contribute an excellent overview of the acute and chronic stress response, and how maladaptive responses result in an allostatic load that is mediated by elevated inflammation. They also address the significance of physical activity as a countermeasure against damaging outcomes of physiological stress.

In the third review, Tian et al. focus on post-traumatic stress disorder as a causal factor in atherosclerosis. They extensively discuss the involvement of different cellular and molecular mechanisms such as glutamate metabolism, ferroptosis, AMPK/mTOR and PI3K-Atk pathways, as well as the role of intestinal microbiota in the development of atherosclerosis, which is often observed in people suffering from PTSD.

In conclusion, the works in this Research Topic illustrate the deep integration of stress into human life and, more importantly, its significance to wellbeing. More and more pathological conditions are attributed not just to the normal aging process but to the inadequate or ineffectual adaptation to the chronic stress of life. In unprecedented times, the significance of the human stress response to known, and, more importantly, new and unknown challenges of human life (e.g., COVID pandemic quarantine, a rising climate crisis) dictates nessessity to recognize and address stress in all aspects of healthcare, education, and work. To close, we want to quote a fitting statement of Dr. Selye that “stress is not something to be avoided,” and send the message that we need to learn how to adapt to new challenges in the new era (Selye, 1956).
